# Supplementary material for: The South-American distribution and southernmost record of Biomphalaria peregrina—a potential intermediate host of schistosomiasis
Source: PeerJ. 2017 May 30;5:e3401. doi: 10.7717/peerj.3401 (PMC5452991; doi:10.7717/peerj.3401)
Supplement: Supplemental Information 1 — Voucher MLP-Ma 14186: partial 16S-rRNA and cytochrome c oxidase subunit I (COI) sequences [file peerj-05-3401-s001.zip › DNA Sequences/GenBank Numbers.pdf]

**Asunto:**	GenBank KY124272-KY124273
**De:**	gb-admin@ncbi.nlm.nih.gov (gb-admin@ncbi.nlm.nih.gov)
**Para:**	robertovogler@fceqyn.unam.edu.ar; robertovogler@yahoo.com.ar;
**Fecha:**	Lunes, 14 de noviembre, 2016 17:26:21

Dear GenBank Submitter:

Thank you for your direct submission of sequence data to GenBank. We have provided GenBank accession numbers for your nucleotide sequences:

BankIt1967775 BMPA3_CO KY124272
BankIt1967775 BMPA3_16S KY124273

The GenBank accession numbers should appear in any publication that reports or discusses these data, as it gives the community a unique label with which they may retrieve your data from our on-line servers. You may prepare and submit your manuscript before your accessions are released in GenBank.

Submissions are not automatically deposited into GenBank after being accessioned. Each sequence record is individually examined and processed by the GenBank annotation staff to ensure that it is free of errors or problems.

You have not requested a specific release date for your sequence data. Therefore, your record(s) will be released to the public database once they are processed. If this is not what you intended, please contact us as soon as possible with the correct release date.

Since the flatfile record is a display format only and is not an editable format of the data, do not make changes directly to a flatfile. For complete information about different methods to update a sequence record, see: https://www.ncbi.nlm.nih.gov/Genbank/update.html

Any inquiries about your submission should be sent to gb-admin@ncbi.nlm.nih.gov

For more information about the submission process or the available submission tools, please contact GenBank User Support at info@ncbi.nlm.nih.gov.

Please reply using the original subject line.
This will allow for faster processing of your correspondence.

Sincerely,

Lori Black, PhD

The GenBank Direct Submission Staff
Bethesda, Maryland USA
****

gb-admin@ncbi.nlm.nih.gov (for updates/replies to GenBank entries)
info@ncbi.nlm.nih.gov (for general questions regarding GenBank)
www.ncbi.nlm.nih.gov/books/NBK51157/ GenBank Submissions Handbook
****
