# Supplementary material for: The South-American distribution and southernmost record of Biomphalaria peregrina—a potential intermediate host of schistosomiasis
Source: PeerJ. 2017 May 30;5:e3401. doi: 10.7717/peerj.3401 (PMC5452991; doi:10.7717/peerj.3401)
Supplement: Supplemental Information 1 — Voucher MLP-Ma 14186: partial 16S-rRNA and cytochrome c oxidase subunit I (COI) sequences [file peerj-05-3401-s001.zip › DNA Sequences/GenBank Submission.pdf]

**Asunto:**	GenBank BankIt submission. Submission ID: 1967775
**De:**	gb-admin@ncbi.nlm.nih.gov (gb-admin@ncbi.nlm.nih.gov)
**Para:**	robertovogler@yahoo.com.ar;
**Fecha:**	Jueves, 10 de noviembre, 2016 19:20:00

Dear Roberto Vogler:

This confirms our receipt of your sequence submission data.
Your submission ID is: 1967775

GenBank accession numbers will be assigned to your sequences within two working days, unless there are issues with your submission that we must ask you to explain first.

Submission to be released immediately after processing

If you have not done so previously and want to download a compressed file of your preliminary submission data, you can do so at https://www.ncbi.nlm.nih.gov/WebSub/

Thank you.

The GenBank Submissions Staff
Bethesda, Maryland USA
****
gb-admin@ncbi.nlm.nih.gov (for replies/updates to records in GenBank)
info@ncbi.nlm.nih.gov (for general questions regarding GenBank)
****
